# Correlation of near‐field optical microscopy and tip‐assisted photoluminescence

**DOI:** 10.1111/jmi.70037

**Published:** 2025-09-27

**Authors:** W. Pfeiffer, N. S. Mueller, R. Hillenbrand, I. Niehues, P. Kusch

**Affiliations:** ^1^ Department of Physics Freie Universität Berlin Berlin Germany; ^2^ Department of Physical Chemistry Fritz Haber Institute of the Max Planck Society Berlin Germany; ^3^ CIC nanoGUNE and UPV/EHU Donostia‐San Sebastián Spain; ^4^ IKERBASQUE Basque Foundation for Science Bilbao Spain; ^5^ Institute of Physics University of Münster Münster Germany

**Keywords:** nanoimaging, photoluminescence, s‐SNOM, TAPL, TMDC

## Abstract

Nanoscale optical imaging has unlocked unprecedented opportunities for exploring the structural, electronic, and optical properties of low‐dimensional materials with spatial resolutions far beyond the diffraction limit. Techniques such as tip‐enhanced, and tip‐assisted photoluminescence (TEPL and TAPL), as well as scattering‐type scanning near‐field optical microscopy (s‐SNOM) offer unique insights into local strain distributions, exciton dynamics, and dielectric heterogeneities that are inaccessible through conventional far‐field approaches, however their combination within the same setup remains challenging. Here we present the realisation of correlative TEPL/TAPL and s‐SNOM measurements within a single side‐illuminated near‐field optical microscope. We address the key experimental challenges inherent to the side‐illumination geometry, including precise laser focus alignment, suppression of far‐field background signals, and the mitigation of competing scattering pathways. Utilising monolayer WSe_2_ as a model system, we demonstrate correlative imaging of material topography, strain‐induced photoluminescence shifts, and dielectric function variations. We visualise nanoscale heterogeneities on a bubble‐like structure, highlighting the complementary information from TAPL and s‐SNOM. This correlative approach bridges the gap between nanoscale optical spectroscopy and near‐field imaging, offering a powerful tool for probing local strain, doping, exciton behaviour, and dielectric inhomogeneities in low‐dimensional materials.

## 1 INTRODUCTION

Optical microscopy with a spatial resolution ranging from tens of nanometres down to the sub‐nanometre region, thus far below the diffraction limit of light, is nowadays routinely provided by advanced tools like scattering‐type scanning near‐field optical microscopy (s‐SNOM), tip‐enhanced photoluminescence (TEPL), and tip‐enhanced Raman spectroscopy (TERS).^1^, ^2^, ^3^, ^4^, ^5^, ^6^ These techniques were successfully used to visualise single molecules, observe propagating polaritons, and image single photon emitters.^7^, ^8^, ^9^, ^10^, ^11^ In most cases, these techniques are realised by illuminating a metallic tip within an atomic force microscope (AFM) with light. This leads to the electromagnetic lightning rod effect and the excitation of plasmons at the tip apex. The combination of both mechanisms generates a strong near‐field between the tip and the sample which is referred to as the nanofocus. The strongly localised near‐field enhances the incoming light, as well as the elastically (s‐SNOM), and inelastically backscattered or emitted light (TERS and TEPL).^12^, ^13^, ^14^ By scanning the tip across the sample and recording the backscattered light, nanoscale‐resolved images are generated, providing additional valuable insights, which are not accessible through conventional far‐field optical techniques. While TEPL reveals electronic and optical properties at the nanoscale and has visualised nanoscale optical heterogeneity in semiconductors and 2D materials,^15^, ^16^, ^17^ TERS enables chemical mapping by detecting vibrational modes.^18^, ^19^, ^20^, ^21^ Recording elastically scattered light within the s‐SNOM in the visible spectral range enables nanoscale imaging of a wide range of material properties, including the dielectric function as well as plasmonic and polaritonic near‐fields.^10^, ^22^, ^23^ It provides insights into the optical properties of materials, identifies doping and strain, and visualises propagating polaritons in real space.^9^, ^10^, ^24^ As s‐SNOM, TERS, and TEPL are based on an AFM, a topography image is recorded simultaneously during nanoimaging.

Initial efforts have been made to integrate s‐SNOM, TERS, and TEPL within a single setup, as they share the same enhancement mechanism, that is, by the metallic tip.^25^, ^26^, ^27^ However, recording simultaneously several signals and correlating the resulting images remains challenging. Reasons are the typical demodulation scheme used in s‐SNOM,^14^ the longer acquisition times in TERS/TEPL (over 100 ms) compared to s‐SNOM (tens of ms), and differences in alignment procedures, which can cause variations in the laser focus position on the tip and sample.

In this study, we employ a commercial s‐SNOM system integrated with a spectrometer (dual s‐SNOM) to enable correlative imaging using near‐field optical microscopy and TEPL. We address the challenges associated with the optical alignment for TEPL in a side‐illumination s‐SNOM configuration and propose potential solutions regarding laser focus, background suppression, and correlation. Additionally, we present several tip‐assisted PL (TAPL) nanoimaging experiments on monolayers of WSe_2_. Note that we use the term TAPL for our experiments instead of TEPL since we observe PL signals influenced by the tip but do not prove pure/ completely background‐free TEPL images. This includes scans over the edge of the material, nanoimaging of a µm‐sized WSe_2_ flake, and the characterisation of a bubble using both TAPL/TEPL and s‐SNOM. By comparing TAPL and near‐field images, we highlight the significance and emerging opportunities of correlative near‐field enhanced nanoimaging within an s‐SNOM system equipped with a visible laser.

## 2 METHODS

### 2.1 FDTD

The finite‐difference time‐domain (FDTD) simulations were performed using the commercial software package Lumerical FDTD Solutions. To simulate the electric fields between the tip and substrate upon illumination we modelled the tip geometry in the 3D software Blender and subsequently imported it in Lumerical. The simulation domain is bounded on all sides by ‘perfectly matched layers’ (PML) which absorb all electric fields and mimic an infinitely extended simulation domain. A Gaussian beam source was placed at the side of the tip and multiple simulations were performed at different positions of the focal point. A mesh override region (with 0.2 nm mesh size) was used to resolve the geometry of the tip apex, such that we obtained accurate electric field values in the small gap between tip and substrate. The dielectric response of the tip and substrate were modelled using the dielectric function of gold determined by Olmon et al.^28^ Electric fields were recorded using a two‐dimensional electric field monitor that cut through the centre of the tip.

### 2.2 s‐SNOM and TEPL setup

**FIGURE 1:**
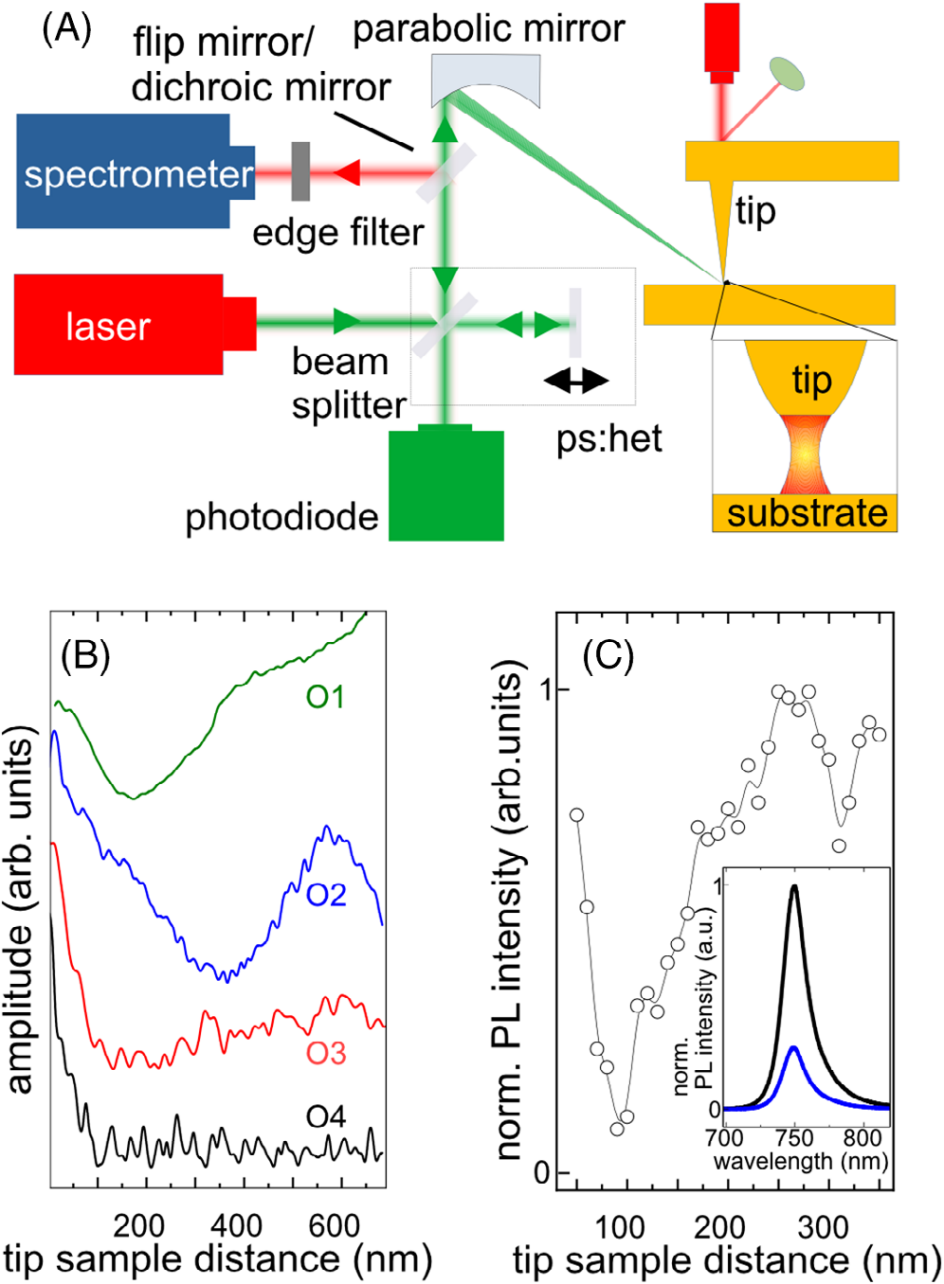
(A) Schematic representation of the dual s‐SNOM setup. The laser beam is directed onto a parabolic mirror, which focuses it onto the tip. The backscattered light is collected by the same parabolic mirror and subsequently focused onto a Si detector using a lens to measure the elastically scattered light. The inelastically scattered light is directed to a spectrometer via a flip mirror (or a dichroic mirror). An edge filter can be applied to suppress the elastically scattered light. (B) s‐SNOM approach curves measured on an Au substrate at a tapping amplitude of 45 nm showing the amplitude of elastically backscattered light as a function of tip‐sample distance, measured at different harmonics. (C) TAPL/TEPL approach curve displaying PL intensity of monolayer WSe_2_ as a function of tip‐sample distance. Inset: PL spectra recorded with the tip close to the sample (black) and at a distance of 100 nm (blue). Taking into account *A*_NF_ = 8·10^2^ nm^2^, the area under the tip, and *A*_FF_ = 2·10^5^ nm^2^, the area of the laser spot, and a ratio of *I*_NF_/*I*_FF_ = 2.6, determined from the PL spectra shown in the inset, the resulting enhancement factor is 650.^24^

We used two specialised scattering‐type scanning near‐field optical microscopes (neaSCOPE from neaspec/attocube systems, see Figure 1A). These instruments operate under tapping mode AFM with standard platinum‐iridium tips and Arrow‐NC Si tips with thermally evaporated 3 nm Cr + 30 nm Au, which have a diameter of 30 nm at the apex (Arrow‐NCPt from NanoWorld), ensuring accurate interaction with the sample. The neaSCOPE systems are equipped either with a normal parabolic mirror (NA = 0.3) or high NA parabolic mirror (NA = 0.7) to focus the laser onto the tip and collect the backscattered light. The tip functions as an optical antenna, converting the incident p‐polarised light into a tightly focused near‐field at the tip apex, often referred to as the nanofocus. This nanofocus interacts with the sample, causing changes in the light scattered from the tip, which encodes the properties of the sample at that location. As the sample is scanned beneath the tip, the scattered light is detected as a function of the sample's position, yielding near‐field optical images with nanoscale resolution, determined by the size of the tip apex and optical near‐field. For examining the local dielectric function of the material under study, the detection of elastically scattered light involves demodulating the s‐SNOM signals at multiples of the frequency of the AFM's tapping mode. For s‐SNOM imaging, tapping mode AFM is applied with an amplitude below 50 nm (specified in figure captions) at a frequency of 267 kHz allowing demodulation of the signal at higher harmonics which, together with pseudo‐heterodyne detection, splits the optical signal into amplitude and phase images that are free from far‐field background artefacts.^29^

The microscope with a normal parabolic mirror utilises a 561 nm laser (Cobolt 06 series, Hübner). The neaSCOPE with a high NA mirror is equipped with a tuneable laser (450–600 nm, C‐WAVE, Hübner) for continuous wave excitation in photoluminescence, Raman, and s‐SNOM studies. The system also includes a grating spectrometer (Andor Kymera 328i) paired with a CCD camera (DU420A‐BEX2‐DD, Andor), facilitating extensive spectral analysis over a broad wavelength range. Operating in side‐illumination, the laser is directed to the tip at an angle of 45°–60°, depending on the parabolic mirror configuration. A flip or dichroic mirror guides inelastically scattered light to the spectrometer (Figure 1A).

**FIGURE 2:**
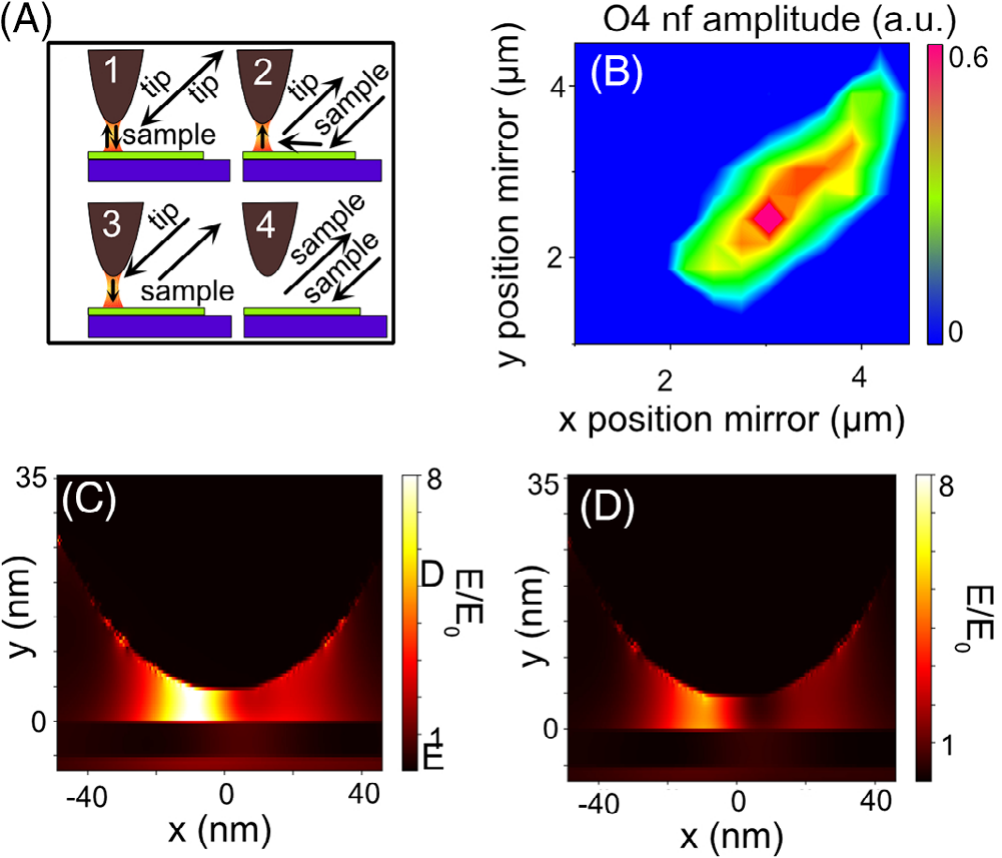
(A) Sketch of the different scattering configurations contributing within the neaSCOPE: A tip 1: T‐S‐T, A tip 2: S‐T, A tip 3: T‐S A tip 4: far‐field scattering. A tip 2 to A tip 4 are background signals that should be suppressed to realise nanoimaging. (B) Near‐field amplitude as a function of laser position in the *x*–*y* plane. First, the *x*, *y*, and *z* positions of the parabolic mirror were adjusted to maximise the amplitude signal. Once the optimal *z* position was determined, it was locked, and the amplitude signal was recorded for varying *x* and *y* positions. The tip position is marked by a circle. The direction of the incident beam is indicated by white dashed lines and an arrow. The maximum amplitude is observed when the tip is slightly offset from the beam centre. (C) FDTD simulation of the local field distribution between a metallic tip and a metal substrate, with the laser focused on the tip shaft. (D) Same as C, but with the laser focused on the tip apex. Cross marks the laser focus position.

**FIGURE 3:**
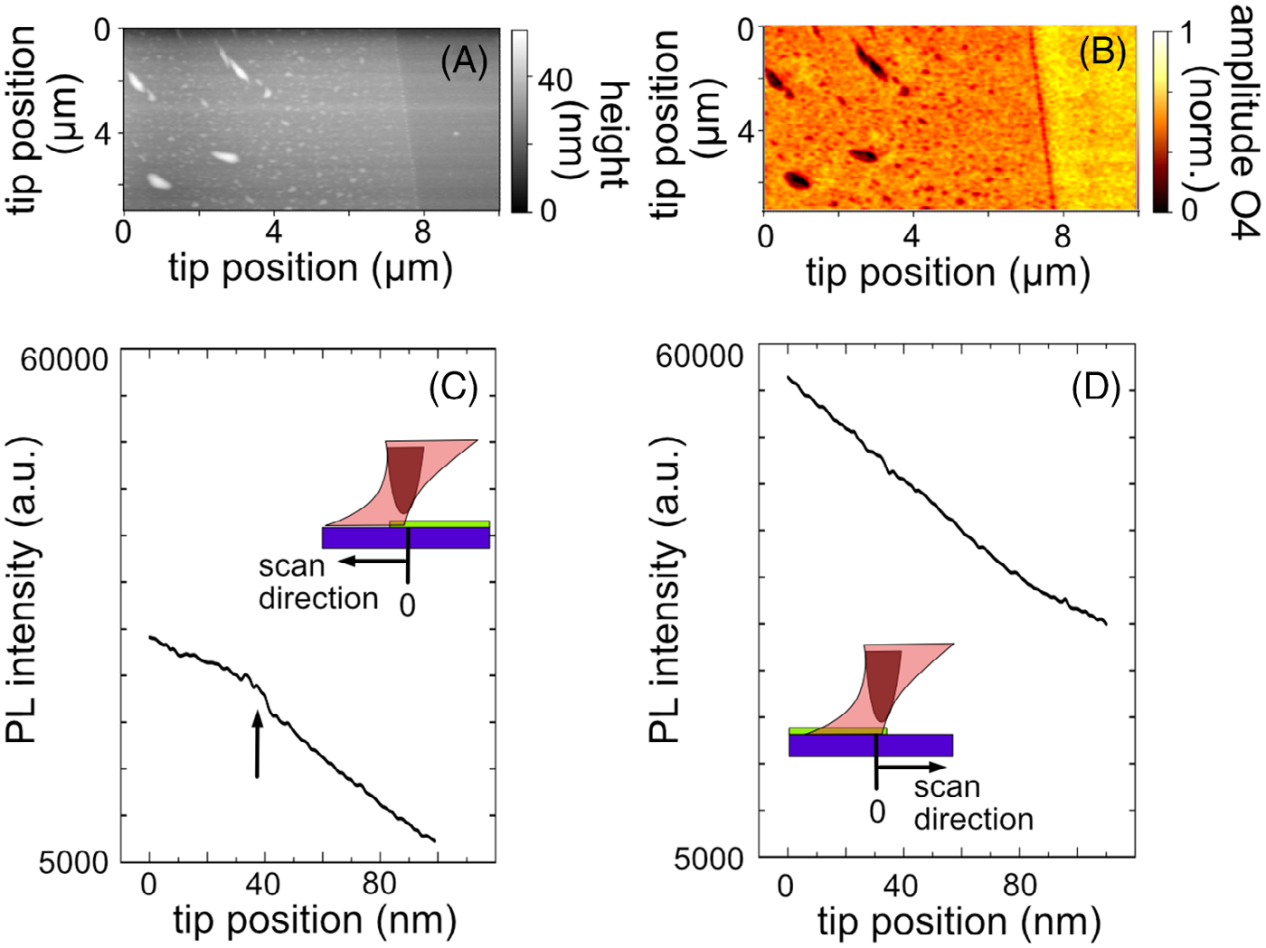
(A) Topography image of a WSe_2_ monolayer on an Au substrate. (B) The corresponding third harmonic amplitude image was recorded at 567 nm laser wavelength, with 40 nm tapping amplitude, at 0.5 mW, and an integration time of 24 ms. (C) Tip‐assisted PL scan (PL intensity as a function of tip position) perpendicular to the sample edge along the white line in Fig. 3A and the edge oriented away from the parabolic mirror. The same parameters are used as in A. The integration time was 0.5 s. (D) The same scan but now with the edge oriented towards the parabolic mirror. Note: For both scans, the tip was placed on WSe_2_ (tip position 0 nm) and scanned towards the edge and finally onto the substrate. The scan direction and 0 position are marked in the insets. Insets: Schematic illustration of sample edge imaged with a defocused beam, with the sample edge oriented toward the parabolic mirror (C), and with the sample edge oriented away from the parabolic mirror (D).

**FIGURE 4:**
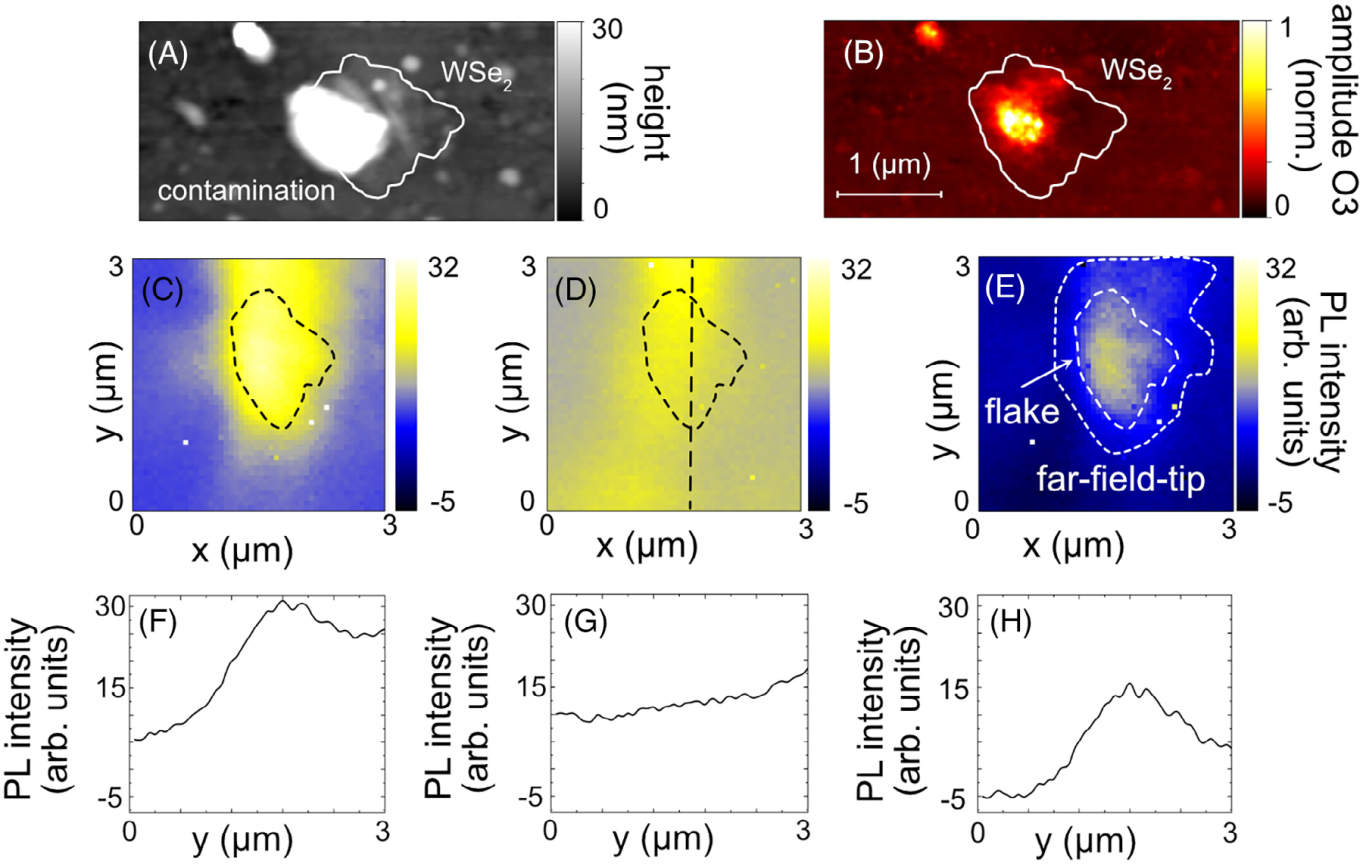
(A) Topography image of a monolayer island of WSe_2_ on an Au substrate. (B) The corresponding near‐field amplitude image recorded at 580 nm laser wavelength, an acquisition time of 19 ms, and a tapping amplitude of 25 nm. (C) Tip‐assisted PL map (PL intensity as a function of tip position) of the WSe_2_ island convoluted with a far‐field background. Measurement with the same parameters as in B. The acquisition time was increased to 0.5 s. (D) PL map at the same area with retracted tip (∼1 µm away). (E) Corrected tip‐assisted PL map of the WSe_2_ island by subtracting the far‐field PL intensities from D and C. To highlight the far‐field subtraction, in F, G, and H, the PL intensity as a function of the tip position along the dashed line is extracted from C, D, and E, respectively.

**FIGURE 5:**
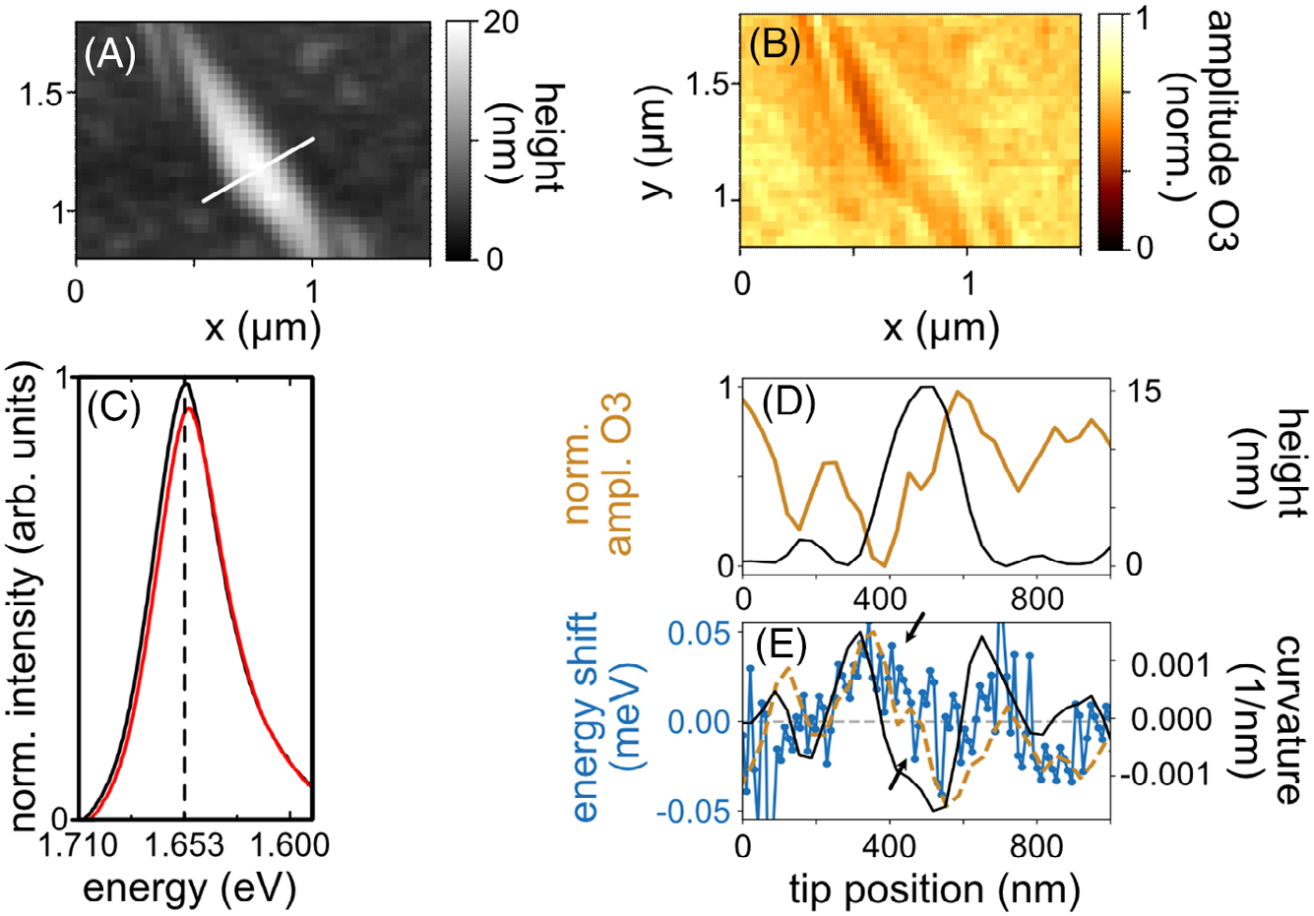
(A) Topography image of a bubble in a WSe_2_ monolayer. (B) Corresponding normalised third harmonic near‐field amplitude image recorded at 561 nm, corrected for tip–sample distance variations. (C) PL spectra recorded on the bubble and on its side, highlighted in E by arrows. (D) Height profile along the white line in A and near‐field amplitude (orange) extracted along the white line in A from B. (E) Excitation energy shift (blue line) extracted from the TAPL spectra shown together with the inverted s‐SNOM amplitude (yellow line, same as in 5D but inverted) and the curvature calculated from the AFM topography (black curve).

PL spectra on the monolayer sample were recorded with tapping amplitudes below 50 nm, laser power of 1 mW illuminating the tip, and an integration time of 1 s for the edge scan, and 0.1 s for the characterisation of the bubble. The obtained spectra are not demodulated and include both near‐field and far‐field contributions. Note: Figures 1, 2, and 4 were acquired with the high NA setup and Figures 3 and 5 with the normal NA neaSCOPE.

### 2.3 Sample preparation

WSe_2_ monolayers were micromechanically exfoliated onto polydimethyldisiloxane (PDMS) from a bulk crystal (hq graphene) and identified by reflection and photoluminescence (PL) microscopy. Subsequently, the atomically thin crystals were stamped onto an Au coated Si substrate. The Au was deposited as a 30 nm thick layer on top of a 4 nm thick Ti adhesion layer via thermal evaporation.

As a substrate for the WSe_2_ island, a 100 nm thick template‐stripped Au film on a glass substrate, attached with UV glue Norland 81 (Thorlabs), was used. The Au was thermally evaporated on an Si wafer and then stripped off with a glass substrate that was glued to the Au/Si. Exfoliated WSe_2_ monolayers were then subsequently transferred onto the Au/glass substrate.

## 3 RESULTS AND DISCUSSION

To achieve correlative imaging by combining PL and s‐SNOM, we employ a neaSCOPE system. This system routinely performs near‐field optical imaging using elastically backscattered light and has recently been adapted for TAPL, TEPL, and TERS by integrating a spectrometer.^25^, ^27^ The setup is shown in Figure 1A and described in the Methods. Optimal performance requires focusing the laser on the metallic tip to maximise the near‐field amplitude, enhancing sensitivity. Achieving spatial resolution below 20 nm involves addressing two key challenges: suppressing far‐field background scattering and isolating tip‐sample‐tip scattered light, where the near‐field enhances both incoming and backscattered light. In side‐illumination s‐SNOM, the ∼1 µm laser spot illuminates the sample, tip, and tip shaft, generating a significant far‐field background. Additionally, further scattering paths may contribute to the background signal that are discussed below. In s‐SNOM the pseudo‐heterodyne (pshet) demodulation scheme suppresses most of the background contributions.^29^

Approach curves, that is, plots of the elastically backscattered light signal as a function of tip‐sample distance at different harmonics when the sample is moving away from the tip, are used to confirm the background suppression, showing signal decay within the first 50 nm, consistent with evanescent near‐field behaviour (Figure 1B).^14^ While the 4th and 3rd harmonics show complete background suppression (near‐field amplitude is close to zero above 50 nm), the 1st and 2nd harmonics exhibit residual background. In the visible spectral range, only higher harmonic near‐field images are considered background‐free and suitable for nanoimaging.

However, in TEPL and TAPL suppressing the background is challenging, as in the used neaSCOPE, no demodulation scheme currently exists. The reason is the combination of a spectrometer with a CCD and the longer acquisition times in the range of milliseconds to seconds. This limitation has significant implications, which is demonstrated by a PL approach curve measured on a WSe_2_ monolayer (Figure 1C), where PL spectra (inset) were recorded as a function of tip‐sample distance. When the tip is close to the monolayer, the PL intensity is high but decays when the sample moves away from the tip to around 100 nm, consistent with s‐SNOM approach curves and tip‐sample‐tip scattering, However, at tip‐sample distances beyond 100 nm, the PL intensity increases and finally exceeds the PL signal observed with the tip close to contact. This increase in PL at large distances may result from reduced shadowing as the tip is away from the sample, increasing far‐field illumination of the WSe_2_. Additionally, when the tip‐sample distance is around 200 nm, far‐field PL form the WSe_2_ monolayer that is scattered and enhanced by the tip's near‐field may contribute to the signal (Note: this is described later as tip‐sample scattering).

Before addressing the challenges of nanoimaging using TEPL within the neaSCOPE and potential solutions, we briefly discuss the main pathways contributing to the measured PL and also within the s‐SNOM. For scattering, the incoming beam excites a localised near‐field at the tip apex that enhances both the incoming and backscattered light by a factor *f*. This is essential for achieving sub‐20 nm spatial resolution (Figure 2A, tip 1) and called tip‐sample‐tip (TST). The second pathway is shown in Figure 2A, tip 2, where the incident beam illuminates not only the tip but also a significant portion of the sample, exciting the sample optically without tip‐enhancement. A fraction of the emitted PL is enhanced by the tip's near‐field, overall leading to a process known as sample‐tip (ST) scattering. Although this enhances resolution beyond the diffraction limit, it remains insufficient to reach the 20 nm resolution and may overshine the TST scattering.^11^ Alternatively, tip‐sample (TS) scattering, Figure 2A tip 3, occurs when the tip's near‐field enhances the incoming beam and excites PL, which emits to the far‐field without further interaction with the tip. ST and TS processes may result from a mismatch between the metallic tip's localised surface plasmon resonance and the strongly red‐shifted PL emission. For instance, in our setup, WSe_2_ is excited at 561 nm, while its PL appears at 782 nm (see Methods). Although ST and TS pathways can achieve 20 nm resolution, their enhancement is significantly weaker than that of TST scattering. Finally, side‐illumination setups also generate direct far‐field PL (Figure 2A, tip 4), where the incident light excites PL that is scattered directly into the far‐field (S), adding an unwanted background signal. Both TEPL and s‐SNOM therefore exhibit four scattering pathways. However, in s‐SNOM, higher harmonic demodulation effectively suppresses the TS, ST, and S pathways, isolating the TST contribution.

In the current neaSCOPE system, all four pathways contribute to the recorded PL.^30^, ^31^, ^32^ For materials with strong Raman scattering or PL, such as graphene and monolayers of transition metal dichalcogenides, this far‐field (S) and ST scattering background can dominate over the TST and TS signal, making nanoimaging very challenging. In contrast, for thin and/or spatially localised samples that have a small volume with a small scattering cross section or a dominant out‐of‐plane optical response, such as p‐NTP molecules or one‐ and zero‐dimensional materials, the background is negligible due to the absence of significant far‐field contributions, making TEPL and tip‐enhanced Raman spectroscopy (TERS) feasible.^26^ Note: In the current version of the neaSCOPE, sample‐tip scattering cannot be suppressed, typically dominates, and leads to lower spatial resolution compared to TEPL, thus, all following images are tip‐assisted (TAPL) and not pure TEPL images.

In s‐SNOM, achieving a strong near‐field is essential for high signal‐to‐noise ratios and effective higher‐order demodulation, which suppresses unwanted background signals. Consequently, precise laser focusing is a critical parameter. Our experimental findings reveal that standard alignment procedures in s‐SNOM often result in a vertically shifted laser focus, positioning it on the tip shaft rather than the tip apex. This is evident in Figure 2B, which shows an image of the s‐SNOM amplitude (4th harmonic) as a function of the laser's *X*–*Y* position, which is controlled through the positioning of the parabolic mirror. The tip is approached to an Au substrate. By first maximising the amplitude signal in *Z* by moving the parabolic mirror (step size between 50 nm and 100 nm) and then scanning the *X*–*Y* plane, we observe an elongated elliptical pattern with the maximum amplitude at the front of the ellipse (the tip is located at the purple area, white circle) – indicating that the laser focus is not on the tip apex. For the gap mode configuration, that is, where a metallic tip is placed on a metallic substrate, a perfectly focused laser would produce a much smaller elliptical pattern with the maximum amplitude signal (tip position) at its centre.

Finite‐difference time‐domain (FDTD) simulations support these findings. Figure 2C presents a simulation of an optimally focused laser for maximum near‐field enhancement. The laser focus was placed on the tip shaft, rather than on the tip apex, generating a strong near‐field signal. In contrast, when the laser is focused directly on the tip apex, the near‐field intensity decreases, Figure 2D. This behaviour is attributed to the lightning rod effect, which enhances the near‐field when the entire tip, rather than just the apex, is illuminated. Therefore, to achieve maximum sensitivity and spatial resolution, the laser in s‐SNOM should be focused on the tip shaft.

In contrast, in TEPL, optimal near‐field enhancement is achieved when the laser is focused on the tip apex, leading to high enhancement factors and a significantly stronger TST signal relative to the background (S and ST).^33^ This fundamental difference indicates that the optimal laser focus position differs for TEPL and s‐SNOM within the neaSCOPE system.

As a result, when using the s‐SNOM alignment for PL measurements, a substantial portion of both the tip and substrate is illuminated generating a strong TS and S background. For strongly photoluminescent 2D systems (e.g., WSe_2_ monolayers) the TS and S background may overwhelm the TST signal, thus masking nanoscale features and preventing nanoimaging. Furthermore, the appearing background arising from far‐field PL depends on the direction of the incoming beam with respect to the sample since it determines the illuminated sample area and therefore the strength of the far‐field PL. As an example, we show imaging of the edge of a WSe_2_ monolayer that is aligned either towards the illumination direction or away from it (Figure 3C and D, for details see insets). For both configurations, we use the s‐SNOM to measure the topography, shown in Figure 3A, and the near‐field optical image, in Figure 3B, to identify the edge. We measure and plot the PL intensity perpendicular to the edge (white line in Figure 3A) as a function of tip position. When the sample edge points away from the illumination direction (Figure 3C), the far‐field contribution is minimised revealing a faint TST intensity increase related to the near‐field contribution (TEPL) on background contribution. The flake edge (indicated by the black arrow in Figure 3C) is clearly visible in PL line scans, though residual slopes in the PL intensity indicate lingering sample‐tip and far‐field contributions. In contrast, when the edge points towards the illumination direction (Figure 3D), a large fraction of the laser illuminates the monolayer on the sample, causing a strong far‐field background S and/or ST background, also emphasised by the higher PL intensity, that masks nanoscale features contained in the TST signal.

As explained before, in the neaSCOPE system, the laser focus is aligned on the tip shaft rather than at the tip apex, complicating the correlation between PL and s‐SNOM images. Since far‐field PL (S) originates from the region where the laser is focused, the spatial origins of PL and s‐SNOM signals differ, leading to a misalignment between the two imaging modalities. To enable the correlation of s‐SNOM and PL and TAPL and also for TEPL, we propose performing the alignment and nanoimaging sequentially within the s‐SNOM system. First, the s‐SNOM part is optimised and a near‐field optical image is recorded. We use a small sample, that is, a WSe_2_ monolayer island with a lateral size on the order of a few micrometres in order to minimise ST and TS scattering. We first adjust the *X*, *Y* and *Z* position of the parabolic mirror to maximise the near‐field amplitude of the s‐SNOM signal and acquire an AFM image along with a near‐field optical image (Figure 3A and B). Once these are obtained, the tip is retracted, and the laser is focused on the WSe_2_ sample. The emitted light is guided to the spectrometer, and the micro‐PL signal is maximised. In micro‐PL measurements, the strongest signal is achieved when the laser is directly focused on the sample. This approach is necessary because the acquisition times for elastically and inelastically scattered light typically differ. After maximising the micro‐PL intensity, the tip is reinserted into the focused laser, and the PL signal is further optimised by fine‐tuning the laser position using the parabolic mirror. In this configuration, the laser is aligned with the tip apex, effectively exciting a near‐field that enhances the photoluminescence (PL) signal. Consequently, the PL signal primarily originates from the region surrounding the tip apex.

To validate that a near‐field is generated and the possibility of nanoimaging via TAPL, we measure an s‐SNOM approach curve and verify that the near‐field signal is present (similar to Figure 1B; note that we use the term TAPL instead of TEPL for this image because, although TEPL is widely used in the literature, we do not explicitly demonstrate pure background‐free TEPL at a resolution of around 20 nm, and want to avoid misrepresentation). Afterward, two maps are acquired: one with the tip engaged, producing a TAPL map (Figure 4C), which still contains far‐field PL, and a second map with the retracted sample (Figure 4D, note that in the neaSCOPE the sample is retracted rather than the tip, as in conventional TERS setups), which captures the far‐field PL. By subtracting the second map (Figure 4D) from the first (Figure 4C), a background corrected TAPL map is obtained (Figure 4E). The WSe_2_ flake is clearly resolved, located at the same position as in the topography and s‐SNOM image (Figure 4A and B). To show the impact of far‐field subtraction, we extract PL profiles (PL intensity as a function of tip position) from Figure 4C and D (along the dashed line), as shown in Figure 4F and G. The resulting subtracted PL profile (Figure 4F–G) is presented in Figure 4H. As evident from the figure, a significant portion of the far‐field PL is effectively removed.

To highlight the potential of correlating TAPL and s‐SNOM within the neaSCOPE by integrating a spectrometer, we directly measure the exciton shift due to strain or the dielectric environment in 2D materials – a capability unattainable with conventional near‐field optical microscopy alone. To demonstrate this, we nanoimage a WSe_2_ flake, featuring isolated bubbles, by AFM and s‐SNOM, Figure 5A and B, respectively. Both the AFM and s‐SNOM nanoimages reveal bubble‐induced topographical (curvature leads to strain) and dielectric changes: both alter the local dielectric function, modulating the near‐field amplitude (Figure 5B). However, s‐SNOM alone cannot quantify strain magnitudes. To address this, we perform TAPL line scans across a bubble (white line in Figure 5A). The surrounding flat WSe_2_ exhibits constant micro‐PL intensity and spectral position, thus only the spectral shift can be directly related with the strain within the bubble. Figure 5C shows exemplary taken PL spectra across the bubble. By correlating the TAPL spectral shift with the tip position, we observe shifts of up to 0.1 meV across the bubble. Using established models,^34^, ^35^ we can calculate a corresponding strain magnitude of 0.002%. Plotting the spectral shift (blue curve, Figure 5E) as a function of tip position and against the curvature calculated from the AFM topography (black curve in Figure 5E) one can see that both curves nicely agree, indicating that strain is indeed the origin of the exciton shift. By comparing with the topography (black curve, Figure 5D), it reveals maximal strain (spectral shift) at the bubble's periphery and negligible strain at its apex, consistent with prior studies.^36^, ^37^

In addition, we can compare the spectral TAPL shift with the near‐field amplitude (yellow curve, Figure 5D). Interestingly, the near‐field amplitude signal is correlated with the spectral PL shift. To highlight this, we plot the inverted near‐field amplitude as a dashed yellow line together with the energy shift. We explain this effect by the following: Strain/curvature induces a shift in exciton resonances, which alters the PL spectral position (A exciton resonance) and modifies the resonances in the dielectric function due to changes in the band structure.^34^ In s‐SNOM measurements performed at 560 nm, this excitation wavelength is slightly above the B exciton resonance in WSe_2_. Tensile strain (positive values) shifts the B exciton to lower energies (redshift), moving it further from the laser energy,^34^, ^35^ thereby reducing the s‐SNOM amplitude. Conversely, compressive strain (negative values) shifts the exciton to higher energies, bringing it closer to the laser energy and increasing the s‐SNOM amplitude. For the PL tensile/compressive strain leads to a redshift/blueshift, leading to the opposite behaviour of the curves. This approach emphasises the complementary capabilities of TAPL and s‐SNOM: Indeed, the recorded TAPL spectra contain far‐field contributions from, for example, sample‐tip scattering, but the correlation of s‐SNOM and PL peak shift enables nanoscale material characterisation, as the photoluminescence (PL) peak position correlates with the near‐field contrast. Furthermore, obtaining strain values is crucial to support and validate finite dipole models used to interpret s‐SNOM data. Such models include the dielectric function, which may be influenced by the strain and thus will lead to slightly different near‐field contrasts.

To achieve TEPL within the neaSCOPE system, it is essential to suppress far‐field contributions and minimise sample‐tip scattering. We propose using high NA parabolic mirrors (approximately NA 0.7) to enhance laser focusing. A demodulation scheme should be implemented to extract the photoluminescence (PL) signal at the tapping frequency, similar to scattering‐type scanning near‐field optical microscopy (s‐SNOM). To that end it would be highly desirable to implement the demodulation scheme for the CCD camera of the spectrometer; however, to the best of our knowledge, this has not yet been realised. Furthermore, the backscattered light should be directed to the spectrometer through a confocal configuration, enabling confocal Raman and photoluminescence (PL) spectroscopy to further suppress far‐field contributions.^38^ Advanced AFM probes which will ensure that the laser focus remains consistent for both s‐SNOM and TEPL measurements could be used to enhance the visibility of the signals. To account for and subtract far‐field contributions while validating the enhanced spatial resolution, a micro‐PL map should be acquired with the AFM tip retracted while keeping the sample in focus. Note: in the new version of the neaSCOPE this option has been implemented.^39^ These optimisations will effectively suppress far‐field PL, reduce acquisition time for PL spectra, and, as we envision, enable simultaneous TEPL and s‐SNOM imaging without realignment, further streamlining correlative nanoimaging.

## 4 CONCLUSION

In conclusion, we have successfully demonstrated the integration of PL and s‐SNOM within a side‐illuminated near‐field optical microscope, enabling correlative nanoscale imaging of optoelectronic and structural properties. By addressing critical challenges inherent to side‐illumination geometries, including laser focus alignment, suppression of far‐field background contributions, and mitigation of competing scattering pathways (e.g., far‐field‐sample vs. tip‐sample‐tip scattering), we established a robust framework for correlative tip‐assisted PL and s‐SNOM nanoimaging. The correlative approach was validated through studies of strained WSe_2_ monolayers, where TAPL provided direct quantification of local strain via spectral photoluminescence shifts, while s‐SNOM concurrently mapped dielectric function variations linked to topography and mechanical deformation. Such findings emphasise the necessity of combining nanoscale spectroscopy with near‐field microscopy to disentangle competing effects (e.g., strain, doping, and dielectric heterogeneity) in low‐dimensional systems. We foresee that the ability to correlate nanoscale optical, topographical, and mechanical properties opens new avenues for studying quantum emitters, exciton dynamics, and strain‐engineered devices.^40^ We envision achieving this by integrating a demodulation scheme, confocal detection, and using tailored AFM tips for imaging, aiming to reduce the acquisition time, and bringing the laser spot position to the same location on the tip for both TEPL and s‐SNOM, being important to extract the TEPL from background (S and ST) signals.
